# Patterns of emergency dispatch calls and their changes during the COVID-19 pandemic in Ulaanbaatar, Mongolia

**DOI:** 10.1186/s12873-025-01273-1

**Published:** 2025-07-07

**Authors:** Gantuya Ganbat, Ayoung Kim, Buyanbilig Namnansuren, Suvd Batbaatar, Ho Kim

**Affiliations:** 1 German-Mongolian Institute for Resources and Technology, Ulaanbaatar, Mongolia; 2 Graduate School of Public Health, Seoul National University, Seoul, South Korea; 3 Graduate School of Life and Environmental Sciences, University of Tsukuba, Tsukuba, Japan; 4 “Ach” Medical University, Ulaanbaatar, Mongolia

**Keywords:** Emergency calls, Temporal analysis, COVID-19 impact, Ulaanbaatar, Mongolia, Emergency medical communication center

## Abstract

**Background:**

Emergency medical dispatch is a crucial component of a national healthcare system. This study provides the first comprehensive analysis of the temporal patterns of emergency medical service evaluations in Ulaanbaatar, Mongolia, and evaluates the impact of COVID-19 lockdowns on those patterns.

**Methods:**

We analyzed emergency medical evaluations recorded by physicians following the dispatch calls received by the Emergency Medical Communication Center in Ulaanbaatar between 2016 and 2021. We assessed trends in the number of emergency calls, demographic distributions, and temporal patterns. Comparative analysis was conducted to assess the impact of COVID-19 lockdowns, with a focus on seasonal, weekly, and hourly variations.

**Results:**

There were 558,457 emergency calls during the study period, and a total of 8 diagnoses (I, J, Y, F, K, G, O, and Z according to ICD-10) constituted 99.1% of the emergency calls. The two most abundant causes for emergency calls in Ulaanbaatar are “Diseases of the circulatory system (I)” and “Diseases of the respiratory system (J)” in all seasons. We analyzed the temporal patterns of the calls and identified that the highest number of calls was received in winter, weekends, and evenings for the total number of calls. Further, we conducted an analysis of calls during COVID-19 strict lockdown periods. A retrospective analysis of the impacts of the COVID-19 pandemic on emergency calls showed that emergency calls decreased by 24% compared to the previous year. Notably, the difference between the COVID-19 strict lockdown period and the preceding period was more pronounced during periods of strict lockdown implementation.

**Conclusions:**

This is the first comprehensive analysis of EMS calls in Ulaanbaatar from 2016 to 2021, providing valuable insights into both long-term temporal trends and the effects of pandemic-related lockdowns. These findings can support improved EMS planning and inform public health policies in low- and middle-income countries.

**Clinical trial number:**

Not applicable.

**Supplementary Information:**

The online version contains supplementary material available at 10.1186/s12873-025-01273-1.

## Background

### Emergency calls

Emergency medical services (EMS) including prehospital treatment and emergency calls are widely known to play an important role in the public health system. Emergency medical conditions frequently occur unexpectedly, such as in cases of injury, sudden illness, complications during childbirth, or imbalances in bodily chemicals. To deal with these emergency medical conditions, EMS aims to address critical medical conditions through quick evaluation, timely application of suitable treatments, and immediate transportation to the nearest suitable healthcare facility using the most effective means available. This approach is designed to improve survival rates, minimize the impact of illnesses, and reduce the risk of long-term disabilities [1]. Information gained from emergency calls serve as important indicators for planning pre-hospital and in-hospital services, professional qualifications in medical services strategies of country.

Although EMS calls can inform ambulance demand management strategies, temporal patterns vary across regions and the reasons and patterns of EMS calls differ between countries [2–4]. Temperoral patterns also differ: Madrid exhibits a bimodal demand pattern [5], London and Madrid see peaks on Fridays and weekends [5, 6], while Singapore peaks on Mondays [7]. These differences highlight the need for context-specific EMS planning.

### Changes in emergency calls during COVID-19

Many countries imposed COVID-19 prevention policies including travel restrictions, social distancing, school closures, stay-at-home requirements, and interruptions of services. The policies and actions influenced emergency calls, emergency department visits, and hospital admissions during the COVID-19 pandemic [8–12]. Specifically, the volume of EMS calls has varied globally. In several locations, EMS calls surged during the first wave of the pandemic. Amiry and Maguire [8] reported a substantial increase in calls of EMS during COVID-19 pandemic. Notable increases were observed in several locations, including Israel (1900%), Copenhagen, Denmark (24%), New York (50%), and Greater Paris, France (225%).

In contrast, studies from other locations reported a decline in EMS calls and emergency department visits. In Finland, the number of emergency department visits and inpatient admissions decreased following lockdown measures [9]. In the United States, weekly emergency department visits were observed to decline up to 45% depending on the region [10]. In Madrid, as well as in Nantes and Lausanne, there was an increase in the number of calls during the first wave of the pandemic [11]. Calls related to stroke and cardiac illness decreased in several U.S. cities [12]. In Melbourne, a strict-lockdown during COVID-19 led to a marked reduction in emergency department visits, likely attributable to the implementation of physical distancing measures [13]. Following the mandate of social distancing measures in Sao Paolo, Brazil, after the onset of the pandemic, the overall number of emergency medical visits decreased, with a significant reduction in non-SARS-CoV-2-related conditions [14]. A similar reduction in emergency department visits due to the pandemic was previously observed during the SARS outbreak in Asia in 2003 [15, 16].

Moreover, the decline in EMS calls extended to cause-specific diseases. D’Ascenzi et al. [17] reported a notable decline in emergency calls for cardiovascular diseases in Tuscany, Italy, during the early days of the COVID-19 pandemic (first quarter of 2020). The reduction in visits and hospitalizations, with a 65% decrease in visits, suggested that patients may have intentionally avoided going to the hospital or contacting EMS. Similarly, Jaffe et al. [18] documented a decrease in calls related to cardiovascular issues, pneumonia, and all injuries for 121-day period from January 1 and April 30, 2020.

Little is known about EMS utilization patterns in low- and middle-income countries (LMICs), including Mongolia. There is a lack of information on how EMS call volumes fluctuate over time and how public health emergencies, such as COVID-19, affect these patterns. This study aims to (1) describe the temporal patterns in EMS evaluations in Ulaanbaatar, Mongolia, and (2) evaluate how these patterns changed during COVID-19-related lockdowns.

## Methods

### Data

Ulaanbaatar, the capital of Mongolia, is home to 1,618,558 people (47.8% of the total population) as of 2023 [19]. Phone number 1-0-3 (medical emergency call number), which is operated by the Emergency Medical Communication Center, receives emergency calls from central six districts (Bayangol, Bayanzurkh, Chingeltei, Khan-Uul, Songinokhairkhan, and Sukhbaatar) of Ulaanbaatar throughout the day. The total population of these districts was 1,446,431 in 2021 [19] and the districts are characterized by a mix of urban development and ger settlements. While wealther areas (such as Khan-Uul and Sukhbaatar) have modern infrastructure, business hubs, and higher living standards, lower-income districts (such as Songinokhairkhan and Bayanzurkh) face challenges like inadequate housing and poor infrastructure.

In this study, an analysis of emergency calls from the Emergency Medical Communication Center in a six-year study period from January 1, 2016 to December 31, 2021 was conducted. The Emergency Medical Communication Center provides medical advice to callers and, when necessary, forwards calls to the nearest hospital for ambulance dispatch and deploys a mobile medical care unit. During each emergency call, the patient’s address, age, and sex are recorded, and the diagnoses are classified according to the International Classification of Diseases (ICD) and registered accordingly. Call duration is not recorded. At present, there is a lack of a comprehensive studies examining the characteristics and patterns of emergency medical calls in Ulaanbaatar. From 2019, onward, the data entry protocol records to include only physician-confirmed cases, ensuring improved data reliability. As such, multiple calls may have preceded a single confirmed cases in the earlier period, and data before 2019 are not directly comparable. Therefore, analyses including those related to COVID-19 are based solely on data from 2019 onwards.

To encourage cross-national comparability in the gathering, processing, categorization, and presentation of mortality statistics, the ICD-10 International Classification of Diseases (ICD) was created. The World Health Organization’s (WHO) relevant edition of the ICD, which contains the selection and modification guidelines, is then used to convert the reported conditions into medical codes. The index states 26 main categories of diseases, etiology, anatomic site, severity, other vital details, and extension [20].

### Setting

In 2020, Mongolia, a lower-middle income country, implemented strict public health measures early in the pandemic, including border closures, school closures, public gathering bans, mask mandates, contact tracing, quarantines, and lockdowns [21]. As COVID-19 was reported and countries started social distancing measures, the Government of Mongolia has implemented interventions accordingly. There are three levels of emergency according to the Law on Disaster Protection [22] - daily preparedness, enhanced readiness, and public emergency readiness. The strictest level of emergency “public emergency readiness” (or strict-lockdown or “stay at home” regime) includes the closing of all levels of schools, partial online work, restricted travel, and public activities. Mongolia has issued the appropriate protection decision in late January 2020, in accordance with the Law on Disaster Protection of Mongolia [22]. The State Emergency Committee, Mongolia, has declared four strict-lockdown periods (a total of 78 days) from November 2020 to April 2021. On November 11, 2020, Mongolia implemented its first strict-lockdown, lasting 33 days.

### Statistical modeling

The study evaluated EMS call patterns and COVID-19 impacts through several analytical approaches:

1. Temporal analysis of EMS patterns: The analysis is based on the emergency call data collected from Ulaanbaatar’s Emergency Medical Communication Center. To evaluate trends in emergency call incidence, the annual incidence rates per 1,000 individuals were calculated by dividing the number of cases by the annual population denominator and compared across different years and regions. Demographic analysis focused on age and gender distributions, with data segmented into age groups to assess call frequency by gender within these cohorts. Furthermore, diagnosis classifications were analyzed, with particular attention given to the most prevalent categories: “Diseases of the circulatory system (I)” and “Diseases of the respiratory system (J)”. During the study period in Ulaanbaatar, data were recorded for 20 out of the 26 main categories of the ICD-10 classification. Diagnosis in categories D, H, U, V, W, and X were not registered during the study period.
2. Evaluation of COVID-19 lockdown impact: To isolate the effect of the COVID-19 pandemic and associated lockdowns. EMS call volumes during the strict-lockdown periods (November 2020 – April 2021) were compared to non-lockdown periods within the same and adjacent years. Descriptive statistics were employed to summarize the data, while graphical representations were employed to illustrate trends.

## Results

### General patterns of emergency calls

Table 1 presents the population of Ulaanbaatar and the emergency calls received by the Emergency Medical Communication Center from 2016 to 2021. In total, *n* = 560,269 emergency calls were received during the study period at the Emergency Medical Communication Center. In total 1,812 fields or less than 0.33% of the total data had incomplete information on address, sex, age, or diagnosis which were omitted from the analysis, leaving 558,457 calls eligible for analysis. The population of Ulaanbaatar increased by 11.8%, while the incidence of emergency decreased by 28.7% over the same period. The highest incidence of emergency call was recorded in 2016, with a rate of 86.1, while the lowest occurred in 2020, with a rate of 54.4. The average number of calls per year (and per day) during the study period was 93,378 (255.6). Between 2016 and 2018, the emergency call incidence rate per 1,000 citizens per year ranged from 75.4 to 86.1. However, this rate decreased to 54.4 to 58.4 in the period from 2019 to 2021.

**Table 1:** The population of Ulaanbaatar and emergency call incidence from 2016 to 2021

	2016	2017	2018	2019	2020	2021
Population of Ulaanbaatar	1,311,251	1,347,598	1,373,150	1,395,773	1,426,645	1,466,431
Emergency call incidence (total 560,269)	112,957	104,086	103,527	81,519	77,594	80,586
Incidence rate of calls per 1,000 citizens per year	86.1	77.2	75.4	58.4	54.4	54.9

The average age of individuals was 53.44 years (Supplementary 1). The most frequently represented age groups were 45–64 years (40%), 25–44 years (26.6%), and those over 75 age groups (14.4%). Conversely, the age group under 15 years was the least represented, comprising only 0.6% of the calls. Gender distribution shows men and women accounted for 36.6% and 63.4% of the calls, respectively. Women generally had a higher frequency of emergency calls compared to men, except for the under-15 age group. Among women, those aged 45–64 made the highest proportion of calls at 24.7%, while women under 15 years made the lowest proportion at 0.35%. Among men, those aged 45–64 represented the largest group, making up 15.3% of the calls, whereas men under 15 years comprised only 0.5% of the calls.

Table 2 presents the categories and the number of calls associated with each. The most common diagnosis, accounting for 69.9% of the total calls, was classified under “Diseases of the circulatory system (I)” followed by “Diseases of the respiratory system (J)” (11.2%), with the “External causes of morbidity (Y)” (6.9%) being the third most abundant diagnosis out of 20 types of diseases. The two most frequent categories is examined in more detail in Supplementary 2.

**Table 2:** Number of recorded diagnoses from 2016 to 2021

Code	A	B	C	E	F	G	I	J	K	L
Incidence of emergency calls	1,822	29	4,313	2,394	12,801	9,347	391,587	62,491	10,494	1,669
Percentage, %	0.3	0.0	0.8	0.4	2.3	1.7	69.9	11.2	1.9	0.3
**Code**	**M**	**N**	**O**	**P**	**Q**	**R**	**S**	**T**	**Y**	**Z**
Incidence of emergency calls	1,449	5	9,330	141	3	5,343	3	587	38,863	7,594
Percentage, %	0.3	0.0	1.7	0.0	0.0	1.0	0.0	0.1	6.9	1.4

### Temporal variations of emergency calls

**Fig. 1:**
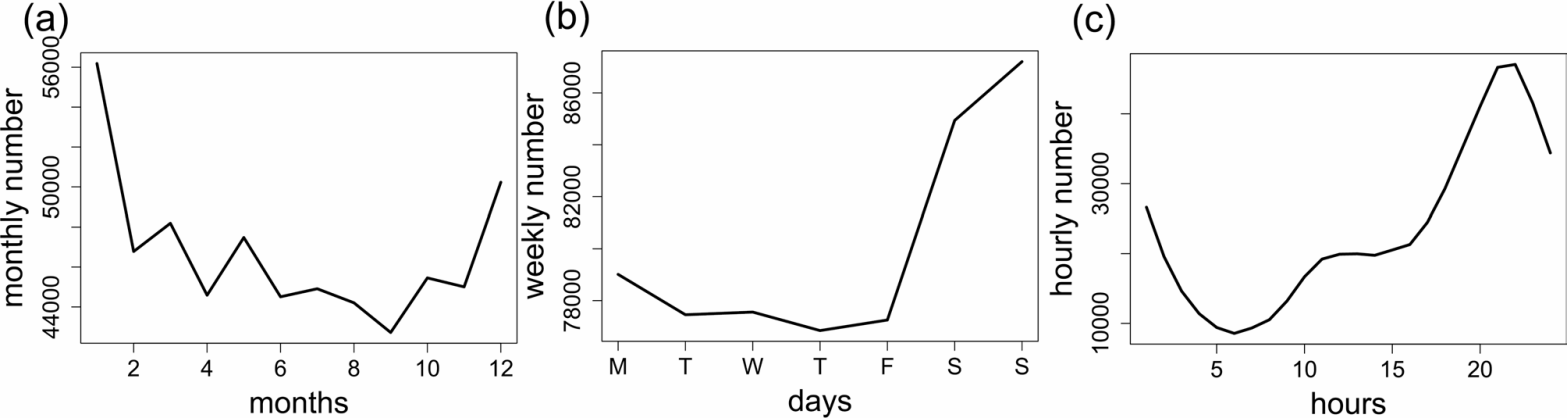
(**a**) Monthly, (**b**) weekly, and (**c**) hourly variations of emergency calls

**Fig. 2:**
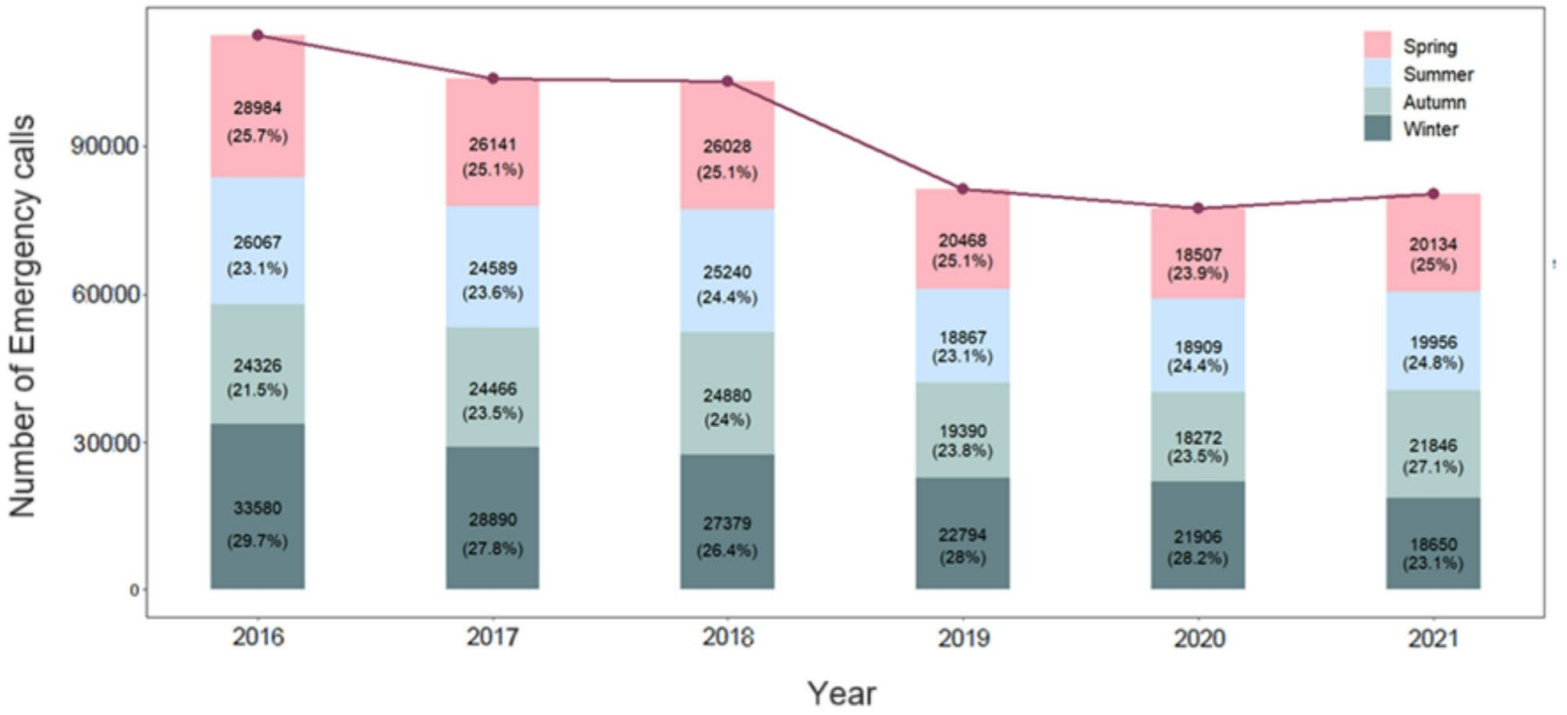
Seasonal number of emergency calls in each year

We examined temporal variations in emergency calls as illustrated in Fig. 1, which displays monthly, weekly, and hourly patterns. The analysis reveals notable temporal variations in emergency call incidence. Generally, the winter months exhibit the largest incidence of emergency calls, while the summer months show the lowest. The seasons are categorized as follows: winter (December, January, February), spring (March, April, May), summer (June, July, August), and autumn (September, October, November). The highest number of emergency calls were received in January, while the lowest number occurred in September, reflecting the distinct seasonal pattern observed throughout the study period, with winter months showing the highest incidence and autumn and summer months exhibiting the lowest (Fig. 2). During the study period, the number of calls was 153,199 (27.3%) in winter, 140,262 (25.0%) in spring, 133,628 (23.8%) in summer, and 133,180 (23.8%) in autumn. In each year, winter months show the highest incidence of emergency calls compared to other seasons, whereas autumn and summer months exhibit the lowest incidence. For instance, the peak in emergency calls from 2016 to 2021 occurred in January 2016, with a total of 12,138 calls, while the lowest incidence was recorded in December 2020, with 5,095 calls. However, the number of calls in each season declined between 2019 and 2021, with a particularly notable decrease during the winter of 2021. In 2021, there were emerging trends of increased emergency calls which was highest in autumn, followed by spring, summer, and winter, making a deviation from the patterns observed in previous years. Additionally, there were some emerging trends of increased emergency calls during the summer and autumn months throughout the 2016–2021 period.

The Emergency Medical Communication Center receives more calls on weekends compared to weekdays, with an average of 77,624 calls during weekdays and 86,074 calls during weekends. Emergency calls exhibit a diurnal cyclical pattern, with the lowest volume occurring around 5 am. The call frequency increases steadily until 10 am, remains nearly stable until 2 pm, and then rises rapidly until reaching a peak between 8 and 9 pm, before gradually declining until 5 am.

### Seasonal pattern for common causes

Between 2016 and 2021, 99.1% (542,507) of the total calls received by the Emergency Medical Communication Center were categorized into eight main ICD-10 categories: I, J, Y, F, K, G, O, and Z. Among these, the highest number of cases were classified into category J, particularly prevalent during the winter season compared to other seasons. Most diseases, with the exception of categories F, K, and G exhibited higher incidence rates in winter and lower rates in other seasons. Table 3 provides a breakdown of emergency calls for the most common diagnosis across each season, indicating that winter calls for category J diseases were 74% higher than those recorded in summer.

**Table 3:** The number of top eight causes of emergency calls by season

Code	Description	Winter	Spring	Summer	Autumn
I	Diseases of the circulatory system (69.9%, *n* = 391,587)	100,712 (26%)	100,465 (26%)	94,606 (24%)	95,808 (24%)
J	Diseases of the respiratory system (11.2%, *n* = 62,491)	22,370 (36%)	13,914 (22%)	12,866 (21%)	13,341 (21%)
Y	External causes of morbidity (6.9%, *n* = 38,863)	14,178 (27%)	12,776 (25%)	13,034 (25%)	11,676 (23%)
F	Mental, Behavioral and Neurodevelopmental disorders (2.3%, *n* = 12,801)	3191 (25%)	3334 (26%)	3159 (25%)	3117 (24%)
K	Diseases of the digestive system (1.9%, *n* = 10,494)	2855 (27%)	2362 (23%)	2915 (28%)	2362 (23%)
G	Diseases of the nervous system (1.7%, *n* = 9,347)	2401 (26%)	2306 (25%)	2410 (26%)	2230 (24%)
O	Pregnancy, childbirth and the puerperium (1.7%, *n* = 9,330)	3556 (38%)	2238 (24%)	1840 (20%)	1696 (18%)
Z	Factors influencing health status and contact with health services (1.4%, *n* = 7,594)	2219 (29%)	1762 (23%)	1736 (23%)	1877 (25%)

### Changes in number of emergency calls during the COVID-19 pandemic

**Fig. 3:**
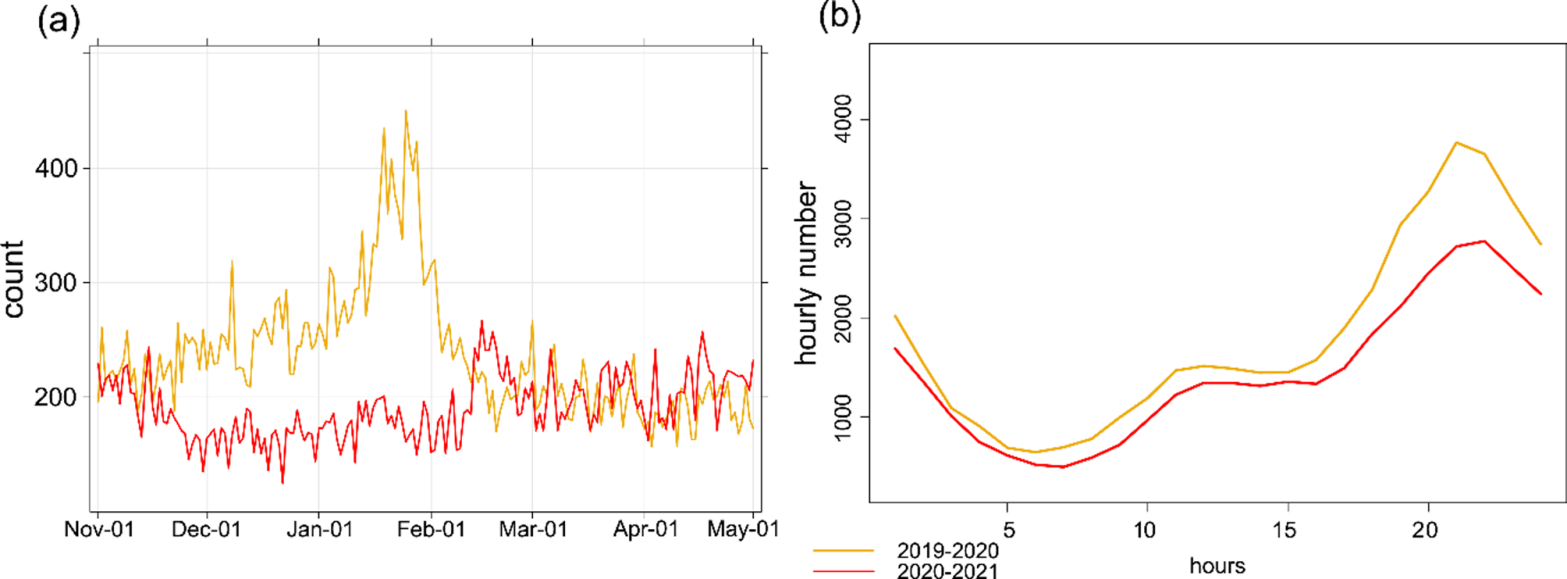
Daily and diurnal emergency call incidences during strict-lockdown and no strict-lockdown periods

The first case of COVID-19 was reported in Wuhan, Hubei Province, China, in December 2019 [23]. The lowest call volumes occurred during the years 2020 and 2021, with average counts reduced (Fig. 3). Monthly trends highlight a notable decline in call volumes from December to February, coinciding with the implementation of strict-lockdown measures. Furthermore, a comparison of hourly trends shows that emergency calls during COVID-19 period were higher than those recorded in the pre-pandemic period. This increase was particularly pronounced during late evening hours (20:00 to 24:00), while the smallest difference was observed during early morning hours (4:00 to 9:00).

**Fig. 4:**
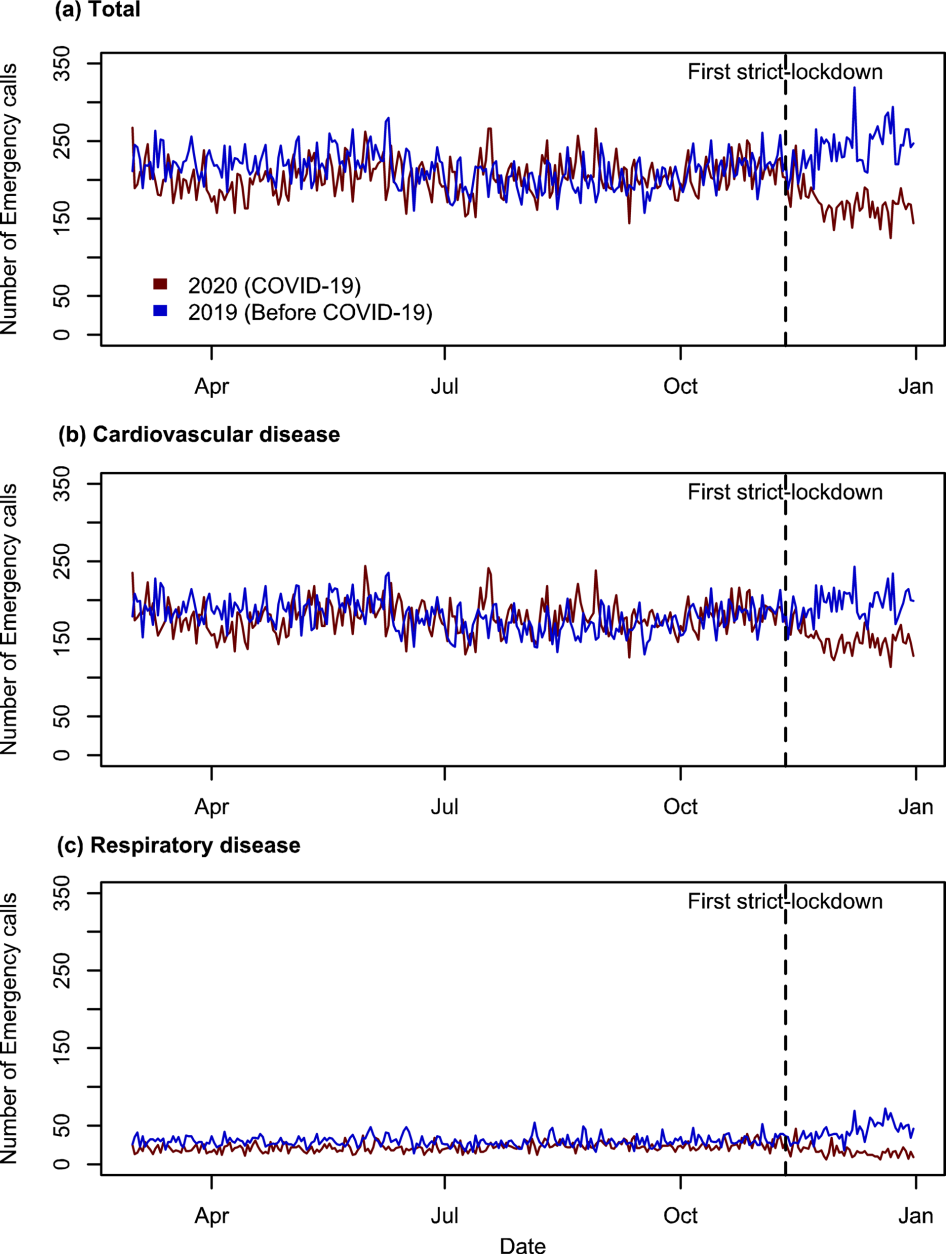
Daily number of Emergency call of (**a**) all, (**b**) cardiovascular diseases (categorized as I with ICD-10 code), and (**c**) respiratory diseases (categorized as J with ICD-10 code) before and after first strict-lockdown (11 November 2020 to 13 December 2020)

Following the imposition of this lockdown, there was a high reduction in the number of emergency calls, though the volume was comparable to pre-lockdown levels (Fig. 4a). Similarly, a corresponding trend was observed for cardiovascular diseases (Fig. 4b); the number of emergency calls declined following the implementation of the first strict lockdown. The lockdown period also had a important impact on emergency calls for respiratory diseases (Fig. 4c).

Between November 2020 to May 2021, four strict-lockdown periods (L1, L2, L3, and L4) were implemented (Table 4). During these full months, the number of emergency calls decreased by 24% compared to the average number of emergency calls recorded during the corresponding periods in the previous year, 2019. The most substantial reduction occurred during L2, which represents the second public strict-lockdown period from December 23, 2020 to January 10, 2021. Notably, following the implementation of the first two strict-lockdown on November 11, 2020, the reduction in emergency calls was more pronounced compared to the subsequent lockdown periods.

**Table 4:** Number of emergency calls during the COVID-19 strict-lockdown periods in Ulaanbaatar

	Period	Details	Daily calls for 2019	Daily calls for 2020–2021	Daily calls for 2020–2021 per 1,000
L1	11 November to 13 December 2020	First strict-lockdown after the first locally-spread case	279.3	174.8 (↓37.4%)	0.12 (↓26.5%)
L2	23 December 2020 to 10 January 2021	Second public strict-lockdown	355.1	169.9 (↓52.1%)	0.12 (↓47.4%)
L3	11–23 February 2021	Third public strict-lockdown	230.8	228.9 (↓0.1%)	0.16 (↓3.0%)
L4	10–25 April 2021	Fourth public strict-lockdown	219.9	215.5 (↓2.0%)	0.15 (↓4.2%)

## Discussion

Emergency calls serve as crucial data source for medical service planning. This study elucidates previously unreported characteristics of emergency dispatch calls in Ulaanbaatar (emergency number 1-0-3). The overall incidence of emergency calls in Ulaanbaatar, Mongolia, is lower compared to other regions such as the Piedmont Region, Italy [24], but higher than in cities like Saitama Prefecture, Japan [25].

These reduction in the number of calls from 2019 onward can be partly attributed to changes in the call registration process (prior 2019, all calls were registered in the system, whereas post-2019, only calls identified by physicians were registered) and the strict-lockdown measures implemented during the COVID-19 pandemic. While the total number of calls decreased from 2019, the proportion of respiratory and circulatory systems increased high. This substantial rise in cases related to respiratory and circulatory systems in 2019 may be partly attributed to changes in patient classification methods, as mentioned. It is likely that diseases other than the specified two categories were not recorded in the data due to the lack of physician confirmation, with symptoms being merely suspected rather than diagnosed.

The analysis identified distinct temporal variations in emergency calls across different seasons, days of the week, and times of day. Emergency calls were most frequent during the winter months, on weekends, and in the evening hours. In Ulaanbaatar, most family health centers operate only on weekdays during regular working hours. As a result, citizens often turn to hospitals for both urgent and non-urgent medical issues during weekends and evenings [26]. A similar trend has been observed in Denmark [27, 28]. In contrast, a study in Singapore indicated different weekly variations, with Mondays being the busiest day and 35.2% non-life-threatening calls occurring on weekends [7]. Moreover, the emergency calls in Ulaanbaatar generally exhibit a singular peak each day in the evening, around 8–9 pm. This pattern is consistent with observations in Western Australia [29] and Saudi Arabia [30]. Additionally, a similar minimum in activity during the early morning hours has also been reported in Madrid [5]. The observed increase in emergency calls during weekends and evenings points to a gap in primary care availability, indicating a need for extended service hours or the establishment of additional after-hours care centers.

Since eight categories (I, J, Y, F, K, G, O, and Z) account for 99.1% of the total calls received by the Emergency Medical Communication Center, it is crucial to develop targeted plans, preparedness strategies, and decision-making processes focusing on these categories. Categories I and J alone constitute 66.5% of the total call volume. In Mongolia, more than half of all deaths are caused by just five diseases, including heart disease and stroke [31]. Many people have risky habits such as smoking—about 25% of the population and half of men smoke daily—poor diets with not enough fruits and vegetables, lack of exercise, and drinking too much alcohol [32]. Nearly half of Mongolians have high blood pressure, and many are overweight or obese and these problems are worse in rural areas where people eat less fiber and more red meat [31]. Because these risk factors are so common, Mongolia has higher rates of heart and lung diseases compared to other countries [33].

Notably, calls for disease category J were 74% more frequent in winter compared to summer. This seasonal variation is primarily attributed to cold weather and air pollution [34]. East Asia and South Asia have some of the highest mortality rates from chronic respiratory diseases, largely due to smoking-related diseases, respiratory viruses, air pollution, and lung diseases [35]. The high volume of emergency calls related to circulatory and respiratory system diseases highlights the importance of strengthening preventive care and implementing targeted public health internventions to improve long-term health outcomes.

A notable decline in emergency calls was observed during the strict-lockdown periods of the COVID-19 pandemic. The highest reduction was evident from December to February, aligning with the implementation of strict-lockdown measures. Mongolia implemented its first strict-lockdown on November 11, 2020, leading to a marked reduction in emergency calls, mirroring global trends observed in other countries. Similar effects of lockdown measures on emergency department visits and emergency calls have been documented globally [8, 36, 37]. Between November 2020 and May 2021, the implementation of four strict lockdowns (L1, L2, L3, and L4) resulted in a 24% reduction in emergency calls compared to 2019, with the most pronounced decrease occurring during L2 (December 23, 2020, to January 10, 2021). The initial lockdowns had a higher impact on reducing emergency calls, but there was a gradual increase in call volume over time. This trend suggests that while strict-lockdown measures were initially effective in curbing emergency call volumes, their effectiveness may have diminished over time due to the prolonged nature of the pandemic and lockdown fatigue, leading to a moderated decline in emergency calls, a pattern consistent with the observations reported by Morello et al. [38]. These findings highlight the need for adaptive public health strategies that balance infection control efforts with ensuring continued access to emergency medical services in Mongolia.

Specifically, emergency calls related to cardiovascular diseases declined during the lockdown. This trend is largely attributed to the heightened anxiety triggered by the onset of the COVID-19 pandemic in early 2020. D’Ascenzi et al. [17] reported a comparable decrease in emergency calls and hospitalizations for cardiac causes in the Tuscany region. They attributed this reduction in healthcare utilization for cardiovascular issues during the lockdown to factors such as fear of COVID-19 infection and misinformation regarding hospital safety. This epidemiological trend highlights the necessity for public health initiatives aimed at reassuring the public about the importance of seeking timely medical care, even amidst pandemic-related concerns.

Similarly, the number of emergency calls for respiratory diseases also diminished. The decline may be explained by increased public vigilance and preventive care measures, driven by heightened awareness of respiratory health during the COVID-19 pandemic [39], with public health measures such as telemedicine campaigns and the mandatory use of face masks playingan instrumental role [40]. Additionally, the closure of schools and the transition to remote learning contributed to decreased viral transmission among children. Furthermore, heightened fear of COVID-19 infection in hospital settings deferred individuals from seeking emergency department care [40].

The shifts in emergency call patterns during the COVID-19 pandemic further emphasize the critical role of maintaining public trust in healthcare systems during crises and ensuring safe, accessible care.

### Strengths and limitations

This study has several notable strengths. First, it is the first to provide a multi-year overview of EMS usage in Ulaanbaatar, offering valuable insight into long-term trends. Second, the use of physician-confirmed EMS records enhance the variability of diagnostic information, minimizing misclassification bias. Third, the analysis spans both pre-pandemic and pandemic periods, allowing for an assessment of how COVID-19 affected emergency care utilization.

However, the study also has several limitations. Changes in the data registration system implemented in 2019 may affect the comparability of data across years. Specifically, call volume data from before 2019 are not directly comparable with data from 2019 onward due to differences in how cases were recorded. To address this, analysis related to changes in call volumes, particularly those examining the impact of the COVID-19 pandemic were limited to comparisons with the year 2019, the most recent pre-pandemic year with a registration procedures consistent with the pandemic period. This approach helps to ensure that observed differences were less likely to result from changes in data collection practices. Additionally, the dataset after 2019 includes only physician-confirmed evaluations, excluding unrecorded or non-dispatched calls, which may underestimate total EMS demand. The absence of clinical outcome data also limits the ability to assess the severity of cases or the effectiveness of interventions. Finally, external influencing factors, such as air quality, temperature, and healthcare access, were not incorporated into the analysis but may have significantly impacted the observed patterns.

### Implications for practice and future research

The insights derived from this research could prove valuable for optimizing emergency services. Specifically, understanding temporal variations in emergency calls can improve resource allocation, staffing schedules, and response efficency. These findings can also inform data-driven policy decisions to enhance emergency preparedness and service delivery. To further enhance health system management, additional research is essential to better understand the characteristics across categories, age dependence, and other critical factors. Moreover, exploring the relationship between air pollution, weather parameters, and the demand for medical care is crucial for gaining a deeper understanding of the factors influencing emergency call volumes and improving medical service delivery.

## Conclusion

This study provides first valuable insights into the patterns of emergency calls in Ulaanbaatar from 2016 to 2021, revealing temporal variations and the impact of the COVID-19 pandemic on call volumes. The findings highlight the importance of developing targeted emergency medical service plans that account for seasonal, weekly, and daily variations in call patterns. Additionally, the study emphasizes the need for adaptive public health strategies during pandemics to ensure continued access to emergency medical care while managing infection control. These insights are crucial for optimizing emergency service delivery and informing public health policies in Ulaanbaatar and similar urban settings.

## Electronic supplementary material

Below is the link to the electronic supplementary material.

Supplementary Material 1

Supplementary Material 2

## Data Availability

The datasets used and/or analyzed during the current study are not publicly available due the patients’ information but are available from the corresponding authors on reasonable request.

## Abbreviations

EMS: Emergency medical services
ICD: International classification of diseases
WHO: World health organization

## References

[1] Kobusingye OC, Hyder AA, Bishai D, Joshipura M, Hicks ER, Mock C. Emergency Medical Services. International bank for reconstruction and Development/The World Bank; 2006. 21250323

[2] Lo SM, Yu YM, Lee LYL, et al. Overview of the Shenzhen emergency medical service call pattern. World J Emerg Med. 2012;3(4):251. 10.5847/wjem.j.issn.1920-8642.2012.04.002. 25215072 10.5847/wjem.j.issn.1920-8642.2012.04.002 PMC4129808

[3] Viglino D, Vesin A, Ruckly S, Morelli X, Slama R, Debaty G et al. Daily volume of cases in emergency call centers: construction and validation of a predictive model. Scand J Trauma Resusc Emerg Med [Internet]. 2017;25(1). Available from: 10.1186/s13049-017-0430-9 10.1186/s13049-017-0430-9 PMC5576313 28851446

[4] Martin RJ, Mousavi R, Saydam C. Predicting emergency medical service call demand: a modern spatiotemporal machine learning approach. Oper Res Health Care [Internet]. 2021;28(100285):100285. Available from: 10.1016/j.orhc.2021.100285

[5] Vargas Román MI, de Miguel ÁG, Garrido PC, et al. Epidemiologic intervention framework of a prehospital emergency medical service. Prehosp Emerg Care. 2005;9(3):344–54. 10.1080/10903120590962157. 16147488 10.1080/10903120590962157

[6] Victor CR, Peacock JL, Chazot C, Walsh S, Holmes D. Who calls 999 and why? A survey of the emergency workload of the London ambulance service. Emerg Med J. 1999;16(3):174–8. 10.1136/emj.16.3.174. 10.1136/emj.16.3.174 PMC1343328 10353041

[7] Ong MEH, Ng FSP, Overton J, et al. Geographic-time distribution of ambulance calls in singapore: utility of geographic information system in ambulance deployment (CARE 3). Ann Acad Med Singap. 2009;38(3):184–91. 10.47102/annals-acadmedsg.v38n3p184. 19347069

[8] Al Amiry A, Maguire BJ. Emergency medical services (EMS) calls during COVID-19: early lessons learned for systems planning (A narrative review). Open Access Emerg Med. 2021;13:407–14. 10.2147/oaem.s324568. 34522146 10.2147/OAEM.S324568 PMC8434918

[9] Kuitunen I, Ponkilainen VT, Launonen AP, et al. The effect of national lockdown due to COVID-19 on emergency department visits. Scand J Trauma Resusc Emerg Med. 2020;28(1). 10.1186/s13049-020-00810-0. 10.1186/s13049-020-00810-0 PMC7716110 33276799

[10] Boserup B, McKenney M, Elkbuli A. The impact of the COVID-19 pandemic on emergency department visits and patient safety in the united States. Am J Emerg Med. 2020;38(9):1732–6. 10.1016/j.ajem.2020.06.007. 32738468 10.1016/j.ajem.2020.06.007 PMC7274994

[11] Navalpotro-Pascual JM, Martin DM, Leon MG, Serrano FN, Blas CA, Isabel BM, et al. Impact of different waves of COVID-19 on emergency medical services and out-of-hospital cardiopulmonary arrest in Madrid, Spain. World J Emerg Med. 2022;13(5):376–89. 10.5847/wjem.j.1920-8642.2022.085. 10.5847/wjem.j.1920-8642.2022.085 PMC9420664 36119777

[12] Sharma R, Kuohn LR, Weinberger DM, et al. Excess cerebrovascular mortality in the united States during the COVID-19 pandemic. Stroke. 2021;52(2):563–72. 10.1161/strokeaha.120.031975. 33430638 10.1161/STROKEAHA.120.031975 PMC7834664

[13] Mitchell RD, O’Reilly GM, Mitra B, Smit DV, Miller JP, Cameron PA. Impact of COVID-19 state of emergency restrictions on presentations to two Victorian emergency departments. Emerg Med Australas. 2020;32(6):1027–33. 10.1111/1742-6723.13606. 32748481 10.1111/1742-6723.13606 PMC7436380

[14] Steinman M, de Sousa JHB, Tustumi F, Wolosker N. The burden of the pandemic on the non-SARS-CoV-2 emergencies: A multicenter study. Am J Emerg Med. 2021;42:9–14. 10.1016/j.ajem.2020.12.080. 33429189 10.1016/j.ajem.2020.12.080 PMC7775794

[15] Man CY, Yeung RSD, Chung JYM, Cameron PA. Impact of SARS on an emergency department in Hong Kong. Emerg Med (Fremantle). 2003;15(5–6):418–22. 10.1046/j.1442-2026.2003.00495.x. 14992054 10.1046/j.1442-2026.2003.00495.x

[16] Chen WK, Cheng YC, Chung YT, Lin CC. The impact of the SARS outbreak on an urban emergency department in Taiwan. Med Care. 2005;43(2):168–72. 10.1097/00005650-200502000-00010. 15655430 10.1097/00005650-200502000-00010

[17] D’Ascenzi F, Cameli M, Forni S, et al. Reduction of emergency calls and hospitalizations for cardiac causes: effects of COVID-19 pandemic and lockdown in Tuscany region. Front Cardiovasc Med. 2021;8. 10.3389/fcvm.2021.625569. 10.3389/fcvm.2021.625569 PMC7994258 33778021

[18] Jaffe E, Sonkin R, Strugo R, Zerath E. Evolution of emergency medical calls during a pandemic – An emergency medical service during the COVID-19 outbreak. Am J Emerg Med. 2021;43:260–6. 10.1016/j.ajem.2020.06.039. 33008702 10.1016/j.ajem.2020.06.039 PMC7318958

[19] Statistics Department of Ulaanbaatar. https://1212.mn/mn/statistic/statcate/573051/table-view/DT_NSO_0300_002V4. Accessed July 15, 2024.

[20] World Health Organization. The ICD-10 classification of mental and behavioural disorders: clinical descriptions and diagnostic guidelines. World Health Organization. 1992.

[21] Chimeddorj B, Mandakh U, Le LV, et al. SARS-CoV-2 Seroprevalence in mongolia: results from a National population survey. Lancet Reg Health West Pac. 2021;17(100317):100317. 10.1016/j.lanwpc.2021.100317. 34841381 10.1016/j.lanwpc.2021.100317 PMC8609908

[22] Law on Disaster Protection. Mongolian Parliament. Enacted June 20. 2003, Ulaanbaatar.

[23] Li Q, Guan X, Wu P, et al. Early transmission dynamics in wuhan, china, of novel Coronavirus–infected pneumonia. N Engl J Med. 2020;382(13):1199–207. 10.1056/nejmoa2001316. 31995857 10.1056/NEJMoa2001316 PMC7121484

[24] Campagna S, Conti A, Dimonte V, et al. Trends and characteristics of emergency medical services in italy: A 5-years population-based registry analysis. Healthc (Basel). 2020;8(4):551. 10.3390/healthcare8040551. 10.3390/healthcare8040551 PMC7763006 33322302

[25] Nakamura A, Manabe T, Teraura H, Kotani K. Age and sex differences in the use of emergency telephone consultation services in saitama, japan: A population-based observational study. Int J Environ Res Public Health. 2019;17(1):185. 10.3390/ijerph17010185. 31888058 10.3390/ijerph17010185 PMC6982294

[26] Tsilaajav T, Ser-Od E, Baasai B, Byambaa G, Shagdarsuren O. Provision of services. In: Kwon S, Richardson E, editors. Mongolia health system review. World Health Organization. 2013.

[27] Møller TP, Ersbøll AK, Tolstrup JS, et al. Why and when citizens call for emergency help: an observational study of 211,193 medical emergency calls. Scand J Trauma Resusc Emerg Med. 2015;23(1). 10.1186/s13049-015-0169-0. 10.1186/s13049-015-0169-0 PMC4632270 26530307

[28] Lehm KK, Andersen MS, Riddervold IS. Non-urgent emergency callers: characteristics and prognosis. Prehosp Emerg Care. 2017;21(2):166–73. 10.1080/10903127.2016.1218981. 27629892 10.1080/10903127.2016.1218981

[29] Turner VF, Bentley PJ, Hodgson SA, et al. Telephone triage in Western Australia. Med J Aust. 2002;176(3):100–3. 10.5694/j.1326-5377.2002.tb04313.x. 11936303 10.5694/j.1326-5377.2002.tb04313.x

[30] Al-Wathinani A, Hertelendy AJ, Alhurishi S, et al. Increased emergency calls during the COVID-19 pandemic in Saudi arabia: A National retrospective study. Healthc (Basel). 2020;9(1):14. 10.3390/healthcare9010014. 10.3390/healthcare9010014 PMC7823911 33374453

[31] Chimed-Ochir O, Delgermaa V, Takahashi K, Purev O, Sarankhuu A, Fujino Y, Naghavi M. Mongolia health situation: based on the global burden of disease study 2019. BMC Public Health. 2022;22(1):5. 10.1186/s12889-021-12070-3. 34983445 10.1186/s12889-021-12070-3 PMC8729000

[32] Global Smoking and Tobaco Harm Reduction Database. https://gsthr.org/countries/profile/mng/1/ Accessed 24 May 2025.

[33] World Health Organization. https://www.who.int/news-room/feature-stories/detail/mongolia-essential-country-specific-tools-to-tackle-ncds? Accessed 24 May 2025.

[34] D’Amato G, Cecchi L, D’Amato M, Annesi-Maesano I. Climate change and respiratory diseases. Europ Respir Rev. 2014;23(132):161–169. Available from: 10.1183/09059180.00001714 10.1183/09059180.00001714 PMC9487563 24881071

[35] Jamrozik E, Musk AW. Respiratory health issues in the Asia–Pacific region: An overview. Respir. 2011;16:3–12. Available from: 10.1111/j.1440-1843.2010.01844.x 10.1111/j.1440-1843.2010.01844.x PMC7192219 20920119

[36] Charlton K, Limmer M, Moore H. Incidence of emergency calls and out-of-hospital cardiac arrest deaths during the COVID-19 pandemic: findings from a cross-sectional study in a UK ambulance service. Emerg Med J. 2021;38(6):446–9. 10.1136/emermed-2020-210291. 33832923 10.1136/emermed-2020-210291

[37] Bekgöz B, Kazan EE, Kahraman AF, Şan İ. Effects of COVID-19 lockdown strategies on emergency medical services. Am J Emerg Med. 2022;60:40–4. 10.1016/j.ajem.2022.06.012. 35905600 10.1016/j.ajem.2022.06.012 PMC9186951

[38] Morello F, Bima P, Ferreri E, et al. After the first wave and beyond lockdown: long-lasting changes in emergency department visit number, characteristics, diagnoses, and hospital admissions. Intern Emerg Med. 2021;16(6):1683–90. 10.1007/s11739-021-02667-2. 33683538 10.1007/s11739-021-02667-2 PMC7938273

[39] Onay ZR, Mavi D, Ayhan Y, Can Oksay S, Bilgin G, Girit S. Did hospital admissions caused by respiratory infections and asthma decrease during the COVID-19 pandemic? Medeni Med J. 2022;37(1):92–8. 10.4274/MMJ.galenos.2022.02779. 35306795 10.4274/MMJ.galenos.2022.02779 PMC8939444

[40] Haklai Z, Applbaum Y, Myers V, et al. The effect of the COVID-19 pandemic on non-COVID respiratory ED visits in Israel. Am J Emerg Med. 2022;53:215–21. 10.1016/j.ajem.2022.01.005. 35074685 10.1016/j.ajem.2022.01.005 PMC8747783

