# Supplementary material for: Patterns of emergency dispatch calls and their changes during the COVID-19 pandemic in Ulaanbaatar, Mongolia
Source: BMC Emerg Med. 2025 Jul 7;25:119. doi: 10.1186/s12873-025-01273-1 (PMC12235795; doi:10.1186/s12873-025-01273-1)
Supplement: Supplementary file 1 — Supplementary Material 1 [file 12873_2025_1273_MOESM1_ESM.docx]

STROBE checklist

| **Section** | **Item No** | **Recommendation** | **Reported on Page No** |
| --- | --- | --- | --- |
| **Title** | 1 | Indicate study design in title or abstract | 1 |
| **Abstract** | 2 | Provide in the abstract an informative and balanced summary of what was done and what was found | 2 |
| **Background** | 3 | Explain the background for the study | 3 |
|  | 4 | State specific objectives and hypotheses | 4 |
| **Methods** | 5 | Data | 4 |
|  | 6 | Setting | 5 |
|  | 7 | Statistical modeling | 5 |
| **Results** | 8 | Report outcome data | 6 |
|  | 9 | Give analysis | 6-8 |
| **Discussion** | 10 | Summarize key results with reference to objectives | 8-10 |
|  | 11 | Strengths and limitations | 10 |
|  | 12 | Implications for practice and future research | 10-11 |
| **Conclusion** | 13 | Supplementary information, Acknowledgements, Funding, Role, Declarations | 11-12 |
|  |  |  |  |
