# Supplementary material for: Patterns of emergency dispatch calls and their changes during the COVID-19 pandemic in Ulaanbaatar, Mongolia
Source: BMC Emerg Med. 2025 Jul 7;25:119. doi: 10.1186/s12873-025-01273-1 (PMC12235795; doi:10.1186/s12873-025-01273-1)
Supplement: Supplementary file 2 — Supplementary Material 2 [file 12873_2025_1273_MOESM2_ESM.docx]

**Supplementary 1**


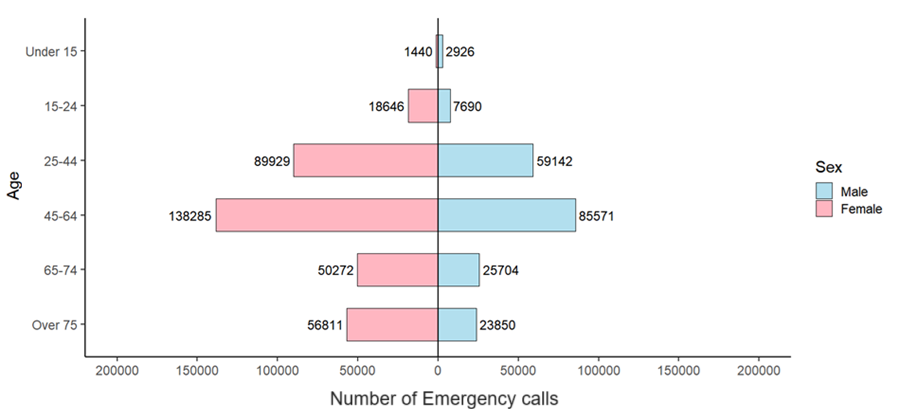


**Supplementary Figure 1.** Age and gender distribution of the total emergency dispatch calls (2016-2021)

**Supplementary 2**

**Detailed analysis on I and J**

Between 2016 and 2018, “Diseases of the circulatory system (I)” and “Diseases of the respiratory system (J)” together accounted for 66.5% of all diseases (Supplementary Table 1). However, their prevalence increased to 98.4%, 98.1%, and 97.6% in 2019, 2020, and 2021, respectively. Although only cases confirmed by doctors were classified as patients beginning in 2019, there was a noticeable increase in the incidence of circulatory system diseases (a 4.36% rise), respiratory system diseases (a 47.51% increase), and both combined (a 9.6% increase) when comparing data from 2019 to 2018.

Moreover, increase in emergency calls related to the two specified disease categories was observed in 2019 compared to 2018. However, in 2020, the number of emergency calls decreased for circulatory system diseases (from 67,108 to 65,269), respiratory system diseases (from 13,118 to 10,838) and the combined total for both categories (from 80,226 to 76,107) compared to the figures recorded in 2019. The phenomenon persisted in 2021, with a continued decrease in emergency calls related to respiratory system diseases (from 10,838 to 8,714) contrasting with an increase in calls for circulatory system disease (from 65,269 to 69,920).

We also analyzed the temporal variations in emergency calls related to circulatory and respiratory diseases, focusing on monthly, weekly, and diurnal patterns as illustrated in Supplementary Figure 2. In terms of monthly variations, the winter months exhibit the highest incidence of emergency calls for both diseases categories. However, the patterns diverge in the remaining months. Notably, emergency calls for circulatory disease experience a decline in February, followed by fluctuations that persist until May. The incidence of emergency calls for circulatory diseases reached its lowest during the summer months, followed by a gradual increase throughout the autumn period. In contrast, emergency calls for respiratory diseases showed a high decline in February, continuing to decrease until reaching their lowest incidence in July, before rising high in December.

In terms of weekly and diurnal variations, both diseases exhibited similar patterns. Emergency calls for both circulatory and respiratory diseases peaked on Saturdays and Sundays, with the lowest frequency observed on Thursdays. Diurnal variations revealed the lowest incidence of calls around 5 am, followed by a steady increase until reaching a peak at 8 pm, after which the call frequency gradually declined for both disease categories.

**Supplementary Table 1** Number of emergency calls on circulatory (I) and respiratory (J) diseases

| **Year** | **I** | **J** | **I+J** |
| --- | --- | --- | --- |
| 2016 | 62,753  (55.6%) | 11,016  (9.8%) | 73,769  (65.3%) |
| 2017 | 62,237  (59.8%) | 9,912  (9.5%) | 72,149  (69.3%) |
| 2018 | 64,304  (62.1%) | 8,893  (8.6%) | 73,197  (70.7%) |
| 2019 | 67,108  (82.3%) | 13,118  (16.1%) | 80,226  (98.4%) |
| 2020 | 65,269  (84.1%) | 10,838  (14.0%) | 76,107  (98.1%) |
| 2021 | 69,920  (86.8%) | 8,714  (10.8%) | 78,634  (97.6%) |


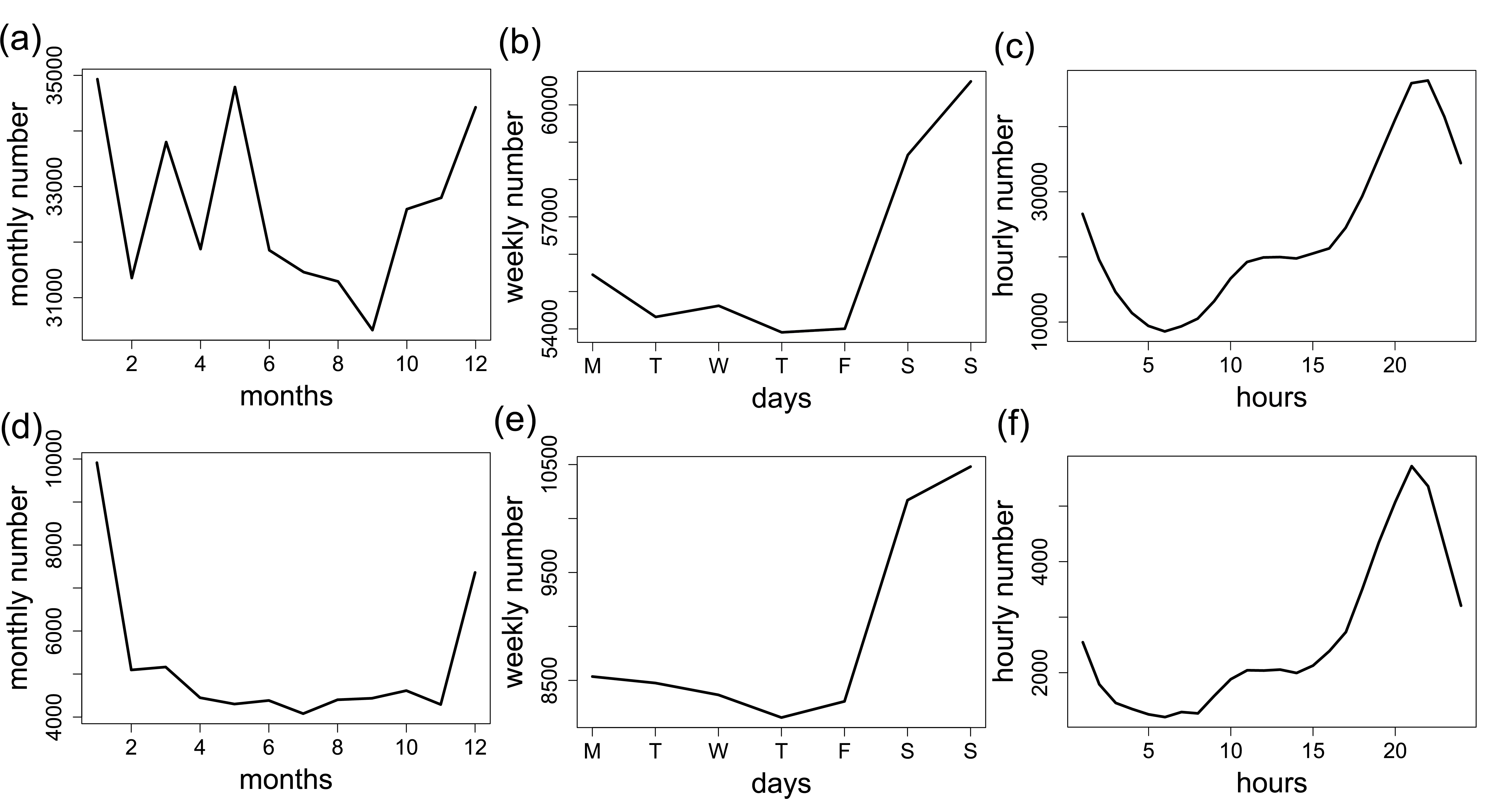


**Supplementary Figure 2.** Age and gender distribution of the total emergency dispatch calls (2016-2021)
